# Body mass predicts isotope enrichment in herbivorous mammals

**DOI:** 10.1098/rspb.2018.1020

**Published:** 2018-06-27

**Authors:** Julia V. Tejada-Lara, Bruce J. MacFadden, Lizette Bermudez, Gianmarco Rojas, Rodolfo Salas-Gismondi, John J. Flynn

**Affiliations:** 1 Department of Earth and Environmental Sciences, Lamont-Doherty Earth Observatory, Columbia University, New York, NY, USA; 2 Division of Paleontology, American Museum of Natural History, New York, NY, USA; 3 Departamento de Paleontología de Vertebrados, Museo de Historia Natural, Universidad Nacional Mayor de San Marcos, Lima, Perú; 4 Florida Museum of Natural History, University of Florida, Gainesville, FL, USA; 5 Zoológico de Huachipa, Lima, Perú; 6 BioGeoCiencias Laboratory, Facultad de Ciencias y Filosofía/CIDIS, Universidad Peruana Cayetano Heredia, Lima, Peru

**Keywords:** isotope fractionation, body mass, stable isotopes, mammals, digestive physiology, sloths

## Abstract

Carbon isotopic signatures recorded in vertebrate tissues derive from ingested food and thus reflect ecologies and ecosystems. For almost two decades, most carbon isotope-based ecological interpretations of extant and extinct herbivorous mammals have used a single diet–bioapatite enrichment value (14‰). Assuming this single value applies to all herbivorous mammals, from tiny monkeys to giant elephants, it overlooks potential effects of distinct physiological and metabolic processes on carbon fractionation. By analysing a never before assessed herbivorous group spanning a broad range of body masses—sloths—we discovered considerable variation in diet–bioapatite δ^13^C enrichment among mammals. Statistical tests (ordinary least squares, quantile, robust regressions, Akaike information criterion model tests) document independence from phylogeny, and a previously unrecognized strong and significant correlation of δ^13^C enrichment with body mass for all mammalian herbivores. A single-factor body mass model outperforms all other single-factor or more complex combinatorial models evaluated, including for physiological variables (metabolic rate and body temperature proxies), and indicates that body mass alone predicts δ^13^C enrichment. These analyses, spanning more than 5 orders of magnitude of body sizes, yield a size-dependent prediction of isotopic enrichment across Mammalia and for distinct digestive physiologies, permitting reconstruction of foregut versus hindgut fermentation for fossils and refined mean annual palaeoprecipitation estimates based on δ^13^C of mammalian bioapatite.

## 1. Introduction

Understanding the structure and history of modern ecosystems and vertebrate communities requires detailed knowledge of extinct and extant animal feeding ecology. This information permits assessment of palaeoclimate, vegetation structure, niche partitioning and predator–prey interactions in fossil ecosystems, so it is crucial to understanding habitat use and trophic specializations over time. Inferences of feeding ecology of extinct vertebrates, however, typically can be inferred only indirectly from morphological data (e.g. dental hypsodonty, enamel microwear and mesowear, cranial shape adaptations, etc.) constrained by correlations between diet and habitats in modern ecosystems. Stable isotope analysis is a powerful tool to reconstruct ancient ecologies and ecosystems, as it is independent of morphology and directly reflects dietary ecology. Because the carbon that animals use to synthesize different tissues relates directly to the ingested food (you are what you eat, [1]), dietary isotopic signatures get recorded in organismal cells and tissues [2–9]. However, during incorporation of carbon from diet to tissue, kinetic isotope effects of an array of physiological processes change the ^13^C/^12^C ratio (*δ*^13^C) of ingested food to that ultimately expressed in tissues (i.e. isotope apparent fractionation, discrimination or enrichment [ɛ*], [3]). For mammalian herbivores, the *δ*^13^C of enamel bioapatite, the most frequently analysed tissue in palaeontology because of its lower susceptibility to alteration during fossilization [10], has been widely assumed to be enriched by approximately 14‰ with respect to dietary *δ*^13^C values, based on a study of modern ungulates and their probable plant foods [3]. This 14‰ value has been systematically but generally uncritically applied in virtually all subsequent dietary and environmental interpretations of fossil and extant mammalian herbivores, regardless of body size, phylogenetic affinities or primary diet source. Intriguingly, though, some studies identified enrichment pattern differences between diet intake and tissues in other mammalian herbivore clades (e.g. [1,4–6]). If these differences are consistent and significant across mammalian clades, size classes or dietary ecologies, they merit concern, as they imply that some previous interpretations of animal ecologies and reconstructed environments could be mistaken.

This study evaluates *δ*^13^C diet–bioapatite enrichment for herbivores across a wide diversity of mammal clades (including non-ungulates and a herbivore group never before assessed—sloths). Isotope enrichment was determined through controlled feeding experiments or constrained diet analyses to test the prevailing assumption that a single enrichment pattern holds for all plant-eating mammals, regardless of life-history attributes or ecology. If it does not, we seek to find and test explanatory variables to predict this value.

## 2. Testing diet–dentition enrichment using sloths, a non-model group of mammalian herbivores

As described in Darwin's ‘Voyage of the Beagle’, fossil native mammals from South America include the ‘strangest animals ever discovered’ [11, p. 80]. More than 170 years later, because of their lack of close modern relatives, ecological analogues or lengthy ghost lineages, the phylogenetic relationships and ecologies of many of these extinct endemic mammal groups remain unresolved. One of the least studied groups of mammals are sloths (Folivora: Xenarthra), although they are crucial to understanding many pre-Pleistocene South American mammal faunal communities given the taxonomic abundance and variety of habitats occupied by this group over the past 35 Myr. Prior interpretations of extinct sloth ecologies relied predominantly on morphology. Consequently, we aim to test those interpretations by means of stable isotope analyses, and provide ecological reconstructions independent of morphology. However, because sloths are outliers in a number of life-history traits characteristic of other mammals [12,13], the standard ungulate-based assumption of a 14‰ diet–bioapatite enrichment [3] is tenuous until directly tested. This caution is particularly warranted because sloths are foregut fermenters, have long food particle mean retention times and produce more methane than other herbivores of similar body masses (the latter experimentally measured in *Choloepus* [13]). Some of these parameters have been suggested as potential drivers of the diet–bioapatite enrichment differences observed in mammals [6]. Thus, determination of the *δ*^13^C diet–bioapatite enrichment 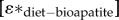 in extant sloths provides a phylogenetically independent test of ungulate-based results in another herbivorous group of mammals. It also becomes indispensable as a first step in assessing isotopic values recorded in extinct sloth bioapatite, by creating a baseline for interpretation of analytical results from fossils.

## 3. Diet–bioapatite *δ*^13^C enrichment in sloths and other herbivorous mammals

Bioapatite is of particular interest because it constitutes the inorganic matrix of bones and teeth, and thus is more likely to be preserved unaltered in the fossil record (especially that from tooth enamel) relative to organic tissues. In addition, the carbonate radical of bioapatite forms from blood bicarbonate, derived primarily from ingested carbohydrates, the main source of carbon in herbivorous diets [5,14]. Bioapatite has proved to be a better predictor of the isotope value of the whole diet than are organic tissues (e.g. collagen, keratin), which mainly track the isotopic composition of the protein(s) used to synthesize them [4,5,14]. Because sloths lack dental enamel, this study analyses sloth dentine bioapatite relative to enamel bioapatite of other mammals. The *δ*^13^C of enamel and dentine bioapatite is considered equivalent if a primary signal is preserved, because any bioapatite (bone, dentine, enamel) is in isotopic equilibrium with blood bicarbonate [15]. In modern specimens, diagenetic alteration is not an issue. A potential problem when dealing with fossil samples is that during taphonomic processes, bone or dentine bioapatite can recrystallize and thus would be susceptible to isotope exchange in this process [16]. For modern bone or dentine, although strong, lengthy laboratory treatments can cause recrystallization (e.g. using 1 M acetic acid to remove secondary carbonates [16–19]), short treatments with weak 0.1 M acetic acid (the protocol used herein, electronic supplementary material) resulted in no detectable change between the *δ*^13^C of enamel and ivory of elephants in protocol tests ([7]; but see [19] for the effect of longer treatments).

We analysed δ^13^C values of dental bioapatite, diet and faecal samples of extant sloths (*Bradypus variegatus* and *Choloepus hoffmanni*) from the Huachipa Zoo (Lima, Peru) kept under controlled and homogeneous feeding conditions (table 1). Because modern sloths vastly underrepresent the ecological and morphological diversity the group had until as late as 10 000 years ago, proper representation of the group in isotopic analyses demands inclusion of extinct representatives. Thus, we analysed dental bioapatite of three Pleistocene ground sloth specimens (*Mylodon darwinii* (Mylodon Cave, Chilean Patagonia) and *Nothrotheriops shastensis* (Gypsum and Rampart Caves)), recovered in association with their dung (table 1). Dung *δ*^13^C is a direct indicator of dietary *δ*^13^C, because there is minimal or no apparent fractionation between diet and faeces 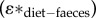 across mammals [8], as corroborated in our analyses of modern sloth faeces (table 1). Collagen atomic C : N ratio was used as a proxy to test for diagenetic alteration of the isotopic composition for Pleistocene sloths (this proxy assesses collagen quality and concentration, which tends to decrease and degrade with the age of the sample [21]). Of the three fossil samples, only *M. darwinii* showed a C : N ratio corresponding to an unaltered sample (C : N = 3.3), and therefore it is the only specimen discussed further. Values of 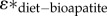 for the other mammals in this study, with either controlled or known diets and associated isotopic values, were gathered from the literature (electronic supplementary material, tables S1 and S2).

**Table 1.** Summary of isotopic analyses: δ^13^C and ε* (diet, dental bioapatite and faeces) for sloths with known diets (through controlled-feeding (*Bradypus*, *Choloepus*) or constrained-diet faeces analyses (^a^*Mylodon*)).

(a) summary of isotopic data of sloths’ diets						
taxa	diet component		δ^13^C_diet component_ (‰)		contribution of each element [δ^13^C (‰)] to final signature of diet^b^	δ^13^C_diet_ (‰)
*Bradypus variegatus*	rubber plant (*Ficus elastica*)		−28.67 ± 1.79		Monospecific diet	−28.67 ± 1.79
*Choloepus hoffmanni*	Purina® DogChow®		−16.5		−1.50	−26.37^b^
	quinoa		−25.8		−2.28	
	broccoli		−27.3		−3.75	
	sweet potato		−27.9		−4.66	
	carrot		−25.3		−4.14	
	spinach		−28.1		−7.29	
	rubber plant		−29.4		−2.75	
^a^*Mylodon darwinii*	calculated from dung		—			−27.17^c^ to −28.14^d^

(b) summary of isotopic data for dental bioapatite and faeces						
	dental bioapatite			faeces		
	*n*	δ^13^C_bioap_ (‰)	ε* (‰)	*n*	δ^13^C_faeces_ (‰)	ε* (‰)
*Bradypus variegatus*	5	−18.65 ± 1	10.31 ± 1.03	5	−28.21 ± 0.53	0.48 ± 0.54
*Choloepus hoffmanni*	10	−14.08 ± 0.66	12.62 ± 0.68	15	−24.98 ± 0.63	1.43 ± 0.64
^a^*Mylodon darwinii*	1	−12.46	15.12^c^ to 16.13^d^	1	−26.7‰	—

^a^recently extinct Pleistocene sloth.

^b^calculation based on a concentration-weighted linear mixing model [20].

^c^assuming diet-faeces enrichment similar to that of *Bradypus*.

^d^assuming diet-faeces enrichment similar to that of *Choloepus*.

Our results (table 1) showed that the three-toed sloth (*Bradypus variegatus*, *n* = 5) has an average *δ*^13^C_bioapatite_ value of −18.65‰ ± 1, which, compared to the *δ*^13^C of its diet at the zoo (−28.67‰ ± 1.79), results in a 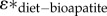 value of 10.31‰ ± 1.03. The two-toed sloth (*Choloepus hoffmanni*, *n* = 10) has an average *δ*^13^C_bioapatite_ value of −14.08‰ ± 0.66, representing a ^13^C-enrichment of 12.62‰ ± 0.68 relative to the *δ*^13^C of its diet (−26.37‰). The *δ*^13^C_bioapatite_ (−12.46‰) of the extinct ground sloth *M. darwinii* is on average 15.63‰ ± 0.51 (15.12‰ to 16.13‰) ^13^C-enriched relative to its diet determined from faeces (−27.66‰ ± 0.49). The high ^13^C-enrichment of *Mylodon*'s bioapatite relative to its diet places this taxon at the uppermost extreme of the 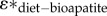 spectrum observed in mammals.

**Figure 1.**
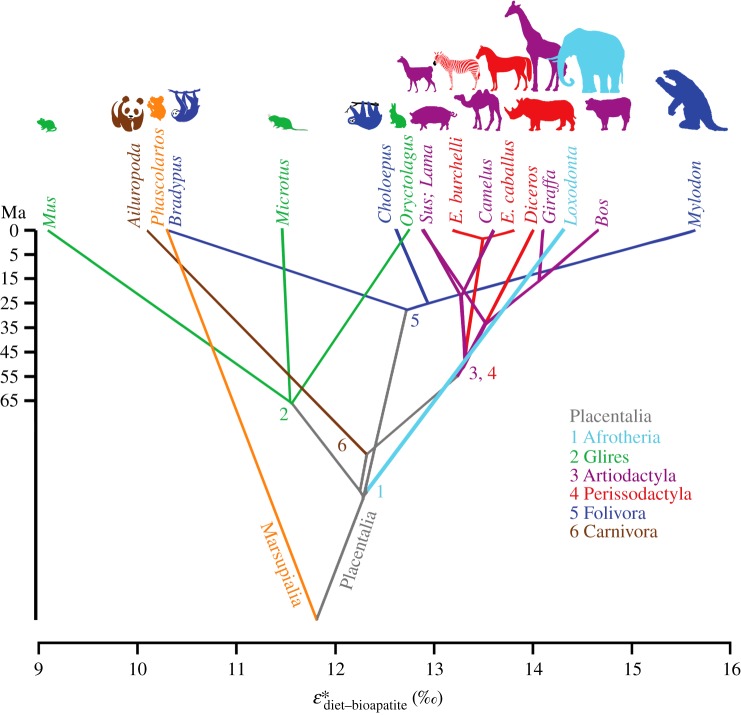
Mammalian phylogenetic tree for sampled herbivores, mapped in a space defined by δ^13^C diet-bioapatite enrichment 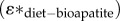. Colours represent clades (see the electronic supplementary material). Note the extremely large range of 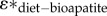 variation in Folivora (sloths), spanning almost the entire spectrum of variation observed in Mammalia. Animal silhouettes are for reference and not to scale.

Placed in a broader mammalian context, results for three sloth taxa span a range of 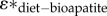 variation (greater than 5‰) never before seen in any mammalian clade (figure 1), encompassing almost the entire range observed across all mammalian herbivores. The range of 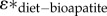 variation in Artiodactyla and Perissodactyla is small (1.2‰ and 1.7‰, respectively). Values of 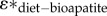 in Artiodactyla range from 12.89‰ in pigs (*Sus scrofa*) to 14.65‰ in cattle (*Bos taurus*), whereas the range of variation observed in Perissodactyla spans from 13.2‰ in the zebra (*Equus burchelli*) to 14.4‰ in the black rhinoceros (*Diceros bicornis*). The range of 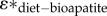 values (across 3.7‰) observed in Glires (i.e. rodents (Rodentia) and lagomorphs (Lagomorpha)) is the second largest after that of Xenarthra. The house mouse, *Mus musculus,* has the lowest 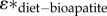 value (9.1‰) of the spectrum of mammals analysed, the prairie vole, *Microtus ochrogaster,* has a 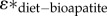 of 11.5‰ and the European rabbit, *Oryctolagus cuniculus,* has a 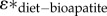 of 12.8‰. Three distantly related taxa: the giant panda, *Ailuropoda melanoleuca* (Carnivora); koala, *Phascolarctos cinereus* (Diprotodontia: Marsupialia); and three-toed sloth (Folivora: Xenarthra), cluster near the lower end of the observed 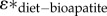 spectrum with values approximately 10–10.5‰.

## Body mass explains high variation of 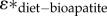 across mammals

4.

**Figure 2.**
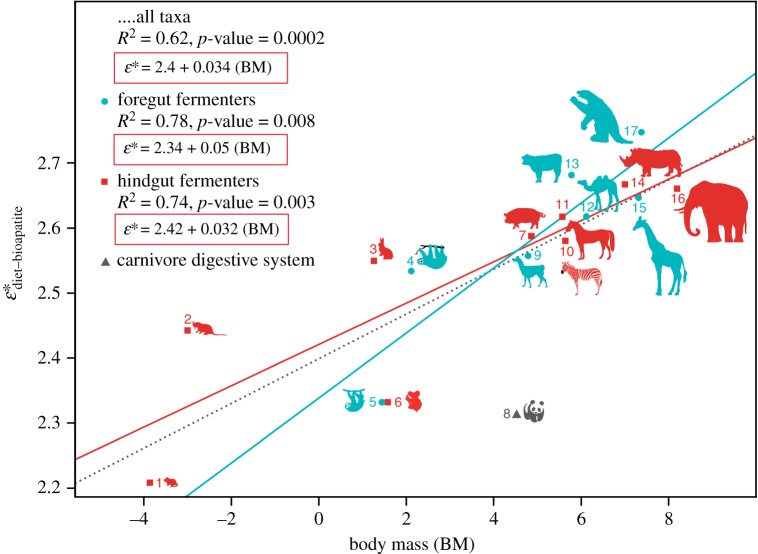
Correlations between body mass and 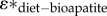 in controlled and constrained diet studies. The regression formula across all mammalian herbivores (dotted line) should be applied when type of fermentation of the herbivorous mammal under study is unknown, or does not fall definitively within foregut or hindgut types of herbivore fermentation (e.g. giant panda). Formulae for foregut (greenish line) and hindgut (red line) fermenters should be applied when the type of digestive fermentation (foregut or hindgut) of the mammal under study is known or probable given its phylogenetic history (see the electronic supplementary material). Species included are: (1) *Mus musculus*, house mouse; (2) *Microtus ochrogaster*, prairie vole; (3) *Oryctolagus cuniculus*, European rabbit; (4) *Choloepus hoffmanni*, two-toed sloth; (5) *Bradypus variegatus*, three-toed sloth; (6) *Phascolarctos cinereus*, koala; (7) *Sus scrofa*, pig; (8) *Ailuropoda melanoleuca*, giant panda; (9) *Lama guanicoe*, guanaco; (10) *Equus burchelli*, zebra; (11) *Equus caballus*, horse; (12) *Camelus bactrianus*, Bactrian camel; (13) *Bos taurus*, cow; (14) *Diceros bicornis*, black rhinoceros; (15) *Giraffa camelopardalis*, giraffe; (16) *Loxodonta africana*, African elephant; (17) *Mylodon darwinii*, giant ground sloth (extinct)*.* All values are in natural logarithmic scale. BM in kg.

**Figure 3.**
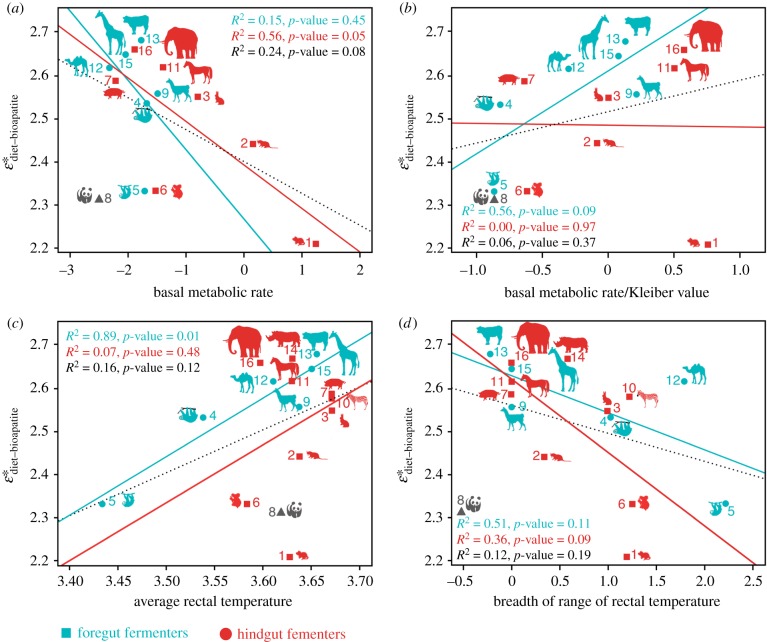
Correlations between 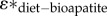 and different life-history traits. (*a*) Basal metabolic rate (BMR). (*b*) BMR, after adjusting for the body mass component of the measured BMR (BMR/Kleiber value). The body mass component was removed by adjusting the BMR to a value corresponding to 3.42*BM^−0.25^ (i.e. the Kleiber value [22]), indicating the scaling of the BMR relative to the body mass for mammals (body mass in grams [23]). (*c*) Average rectal temperature and (*d*) breadth of range of rectal temperature. All values in natural logarithmic scale. Temperature in °C. Dotted black line is the regression model for all taxa included; the red and greenish lines are the regression models for hindgut and foregut fermenters, respectively. Numbers for taxa same as in figure 2.

Aiming to find explanatory variables correlated to the broad range of 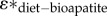 values now observed across mammals with controlled diets or well-known *δ*^13^C values of diets in the wild (even among three members of only one clade, sloths), a series of physiologically correlated parameters were regressed against 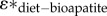: body mass, phylogeny, basal metabolic rate (BMR), BMR corrected for known covariance with body mass (BMR/Kleiber value), average body temperature (rectal temperature) and breadth of body temperature variation (figures 2 and 3). Information for other parameters potentially relevant to understanding these 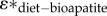 differences (e.g. nutritional quality of diet, food particle mean retention time, CH_4_ yield, diversity and abundance of digestive tract microorganisms) were not available for most taxa with known values of 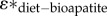.

Body mass shows a strong and significant positive correlation with 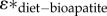 (*r*^2^ = 0.62 (all taxa included), *p*-value = 0.0002; figure 2) across mammalian herbivores. Correlation between body mass and 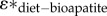 is even stronger if mammals are separated by digestive system type: foregut fermenters (*r*^2^ = 0.78, *p*-value = 0.008) versus hindgut fermenters (*r*^2^ = 0.74, *p*-value = 0.003) (figure 2). We tested for and rejected the possibility that the significance of the regression was driven by the effect of the outliers (particularly those of small size), by performing quantile and robust regression model analyses (table 2). Regression diagnostics identified the vole, panda and koala as outliers, but only the vole influenced the regression (Cook's *D* > 0.5). Its exclusion in a secondary analysis, however, did not significantly alter the coefficient values; in fact, it makes the relationship even stronger (table 2). Based on a reduced taxon dataset (i.e. excluding taxa with unknown values, such as *Mylodon*, *Diceros* and *Equus burchelli*), we also performed generalized linear model analyses based on Akaike information criterion (AICc) tests, to assess whether a single variable, reduced model or various model subsets were better supported by the data than a complete global model that includes all the potential predictors (table 2). Three models were well supported (delta AICc < 2), all three of which included body mass as the most important predictor variable, with the model including only body mass selected as the best model (table 2).

**Table 2.** Summary of quantitative analyses. ((*a*) Summary of coefficients and significance values for linear regressions performed. Quantile regression estimates the conditional median of the response variable, unlike ordinary least squares (OLS) that estimates the approximate conditional mean. Robust regression fittings (objective functions used were Huber and Tukey bisquare) are performed by iterated, re-weighted least-squares analyses. The formulae proposed use the average of the coefficients given by the different regression models (see the electronic supplementary material). (*b*) Component models (delta AICc < 2) that best predict δ^13^C diet–bioapatite enrichment 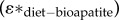 in herbivorous mammals given current data. Body mass alone is the best predictor of enrichment. Other, more complex models that simultaneously assess more potential influences, do not significantly improve prediction of the enrichment in the AICc analyses. Abbreviations: d.f., degrees of freedom; logLik, maximum log-likelihood; AICc, Akaike information criterion corrected for small sample sizes; delta, difference in AICc between the current and the best model; weight, prior weights used in model fitting.)

(*a*) linear regressions	intercept	slope	*R*^2^	*p*-value	
**all taxa included**					
OLS model fit	2.4	0.034	0.62	0.0002	
OLS model fit excluding outliers	2.39	0.04	0.83	5.35×10^−6^	
quantile regression fit	2.37	0.041			
robust regression (Huber estimator)	2.4	0.036			
robust regression (Tukey bisquare estimator)	2.4	0.035			
average	2.39	0.037			
**hindgut fermenters**					
OLS model fit	2.42	0.032	0.74	0.003	
OLS model fit excluding outliers	2.4	0.036	0.89	0.001	
quantile regression fit	2.48	0.022			
robust regression (Huber estimator)	2.43	0.03			
robust regression (Tukey bisquare estimator)	2.51	0.019			
average	2.45	0.028			
**foregut fermenters**					
OLS and robust model values	2.34	0.05	0.78	0.008	
quantile regression fit	2.24	0.07			
robust regression (Huber estimator)	2.34	0.05			
robust regression (Tukey bisquare estimator)	2.34	0.05			
average	2.34	0.06			

(*b*) component models	d.f.	logLik	AICc	delta AICc	weight
body mass alone	3	12.71	−17.02	0.00	0.52
body mass + average rectal temperature	4	14.03	−15.62	1.41	0.26
body mass + basal metabolic rate	4	13.91	−15.38	1.64	0.23

Comparing different models of trait evolution, 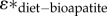 data best fitted a white noise model (lowest AICc, electronic supplementary material, table S3), which assumes data come from a single normal distribution with no covariance structure among species, indicating no phylogenetic signal in the observed values of mammalian 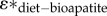. No significant correlation was found between 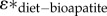 and BMR for all mammalian herbivores, nor for any analysis of BMR after its body mass component was removed (i.e. the Kleiber value, or the ¾ power to which the BMR has been described to scale relative to body mass in mammals, [22]). There is a significant correlation of the raw BMR for hindgut fermenters only, although the lack of significance for even hindgut fermenters in the BMR/Kleiber analysis indicates that body mass explains that correlation (figure 3*a*,*b*). Although no correlations were found between 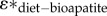 and breadth of rectal temperature variation, a strong significant correlation was identified between 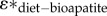 and average rectal temperature, but for foregut fermenters only (*r*^2^ = 0.89, *p*-value = 0.01) and not for hindgut fermenters or all mammalian herbivores (figure 3*c*,*d*). However, a regression analysis of average rectal temperature and body mass reveals that this correlation in foregut fermenters also is not independent of body mass (electronic supplementary material, figure S1). Rates of metabolic biochemical reactions are controlled by temperature [24], but based on our dataset, analyses and tests, body temperature variables do not seem to play a role in mammalian patterns of carbon isotopic fractionation *independently* of body mass. Analytical experiments to propose and test specific mechanistic explanations for how body temperature, independent of or in association with factors driven by body mass, might affect 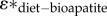 are outside the scope of the present contribution, but deserve future exploration for a better understanding of 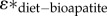 variation in mammals. Overall, body mass is the best predictor of 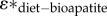 (lowest AICc, table 2), and it is the only parameter that significantly correlated with 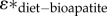 in all cases (i.e. when all taxa were included, as well as when mammals were separated by digestive physiology into foregut and hindgut fermenters; electronic supplementary material, table S4). This newly recognized body-size dependence of carbon isotope enrichment from ingested diet to bioapatite tissue formation requires application of size-calibrated 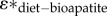 calculations, rather than a single enrichment value across all mammalian herbivores (proposed formulae in figure 2 and the electronic supplementary material), and potential reinterpretation of some prior dietary and ecological results dependent on a single value of approximately 14‰. Additional future controlled-feeding experiments across a large range of body sizes for both foregut and hindgut fermenters would permit refinement of the regression formulae for the correlations between body mass and 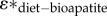.

The *δ*^13^C enrichment that occurs between dietary input and metabolic CO_2_ (that supplies the dissolved inorganic carbon in blood, in isotopic equilibrium with bioapatite) has been attributed to digestive physiology [6], in particular methane (CH_4_) production [25]. Methane, ^13^C-depleted by almost 40‰ relative to the ingested diet, is a by-product gas during CO_2_ reduction [26], which in mammals is owing to microbial activity during digestive processes [25]. As a consequence, and acknowledging that attribution of methane production as the principal driver of the 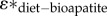 differences observed in mammals remains only theoretical (but see [25] and [26]), one would expect herbivores producing large amounts of methane to also show large 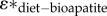 values, as has been experimentally demonstrated in cattle [6,25]. The magnitude of microbial activity in the digestive tract, expressed as biochemical transformations leading to differential diet-tissue enrichment values (ɛ*), is predicted to result from the interaction of variables such as the capacity of the fermentative vat, amount and diversity of microorganisms living in it, and metabolic activity of the microorganisms [27], as well as the particle mean retention time [13] and the quality and quantity of the host's diet [28]. Comprehensive understanding of intermediary metabolic processes of intestinal microorganisms, and related isotopic fractionation pathways and patterns, including other potential mechanisms for sequestering light isotopes, however, is lacking for most vertebrate groups.

One taxon with a much lower than expected 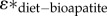 given its body mass was the giant panda (*A. melanoleuca*). The substantial deviation from the predicted 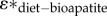 value probably is because the giant panda retains a carnivore-like gastrointestinal tract reflective of its ancestry as a member of Carnivora, in spite of its evolution of an exclusively herbivorous, bamboo-specialist diet. Indeed, because of its long midgut but simple stomach, the giant panda lacks a digestive strategy that allows cellulose breakdown by means of the extended periods of gastric fermentation that is typically found in highly methanogenic mammalian herbivores. Furthermore, the giant panda's gut microbiota diversity is low and of a phylogenetic composition markedly different from that of other herbivores (i.e. lacking cellulose-degrading phylotypes), which is attributed as being responsible for its meagre digestive efficiency [29].

The house mouse was the smallest (approx. 20 g) mammal evaluated, and its 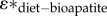 was lower than expected for its body mass. It is noteworthy that the other rodent analysed, the prairie vole (approx. 50 g), also deviates from the regression model but instead exceeds the expected value. A comparative study of the fermentative structures (i.e. caecum + colon in hindgut fermenters) shows that the absolute and relative size of these structures in voles each exceeds (by more than 2 and 3 times, respectively) that of other rodents that are even two orders of magnitude larger in absolute body size [28]. In voles, this relatively large gut capacity greatly increases the space available for microbial food fermentation, and might account for their higher than expected 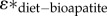 value relative to body size. Furthermore, voles possess a superior digestive efficiency relative to other rodents, correlated with a greater dry matter digestibility and selective retention of fine particles [30]. Finally, in addition to possessing considerably smaller fermentative vats, mice need relatively higher rates of food turnover given their relatively higher metabolic requirements, leaving less time for food fermentation and therefore CH_4_ production. The type of food selectivity (e.g. strict herbivory versus omnivory) might also play a role in controlling 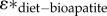 variation. Comparative studies of guinea pigs (herbivore) and rats (omnivore) show lower methane production in the latter, explained as due to a lesser dependence on microbial fermentation owing to their generally omnivorous diets [31]. A comparable explanation may be applicable to the third Glires species analysed, the European rabbit (Lagomorpha), which like the vole has a higher than expected 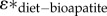 value relative to its body mass, but a smaller residual from the overall regression than that of the vole. However, because the calculation for the rabbit was based on only one specimen [6], more data are needed to rule out the possibility that the higher than expected 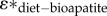 had been overestimated.

Besides the mouse and giant panda, the other two mammals with substantially lower 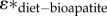 relative to body mass were the three-toed sloth and koala, which share similar body masses (approx. 4 kg), low metabolic rates and strict folivory. Because the quality of ingested food is thought to influence microbial gut population activity [28], the additional metabolic challenges imposed by strict folivory (e.g. high content of secondary toxic compounds, low fibre content) would be expected to play a role in the 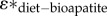 observed in those taxa. In addition, both the three-toed sloth and koala feed on a small number of plant species. Koalas feed preferentially on *Eucalyptus* spp. [32], whereas three-toed sloths eat *Cecropia* and *Ficus* spp. and in general are highly selective in their food choice, avoiding leaves of other tree species that are only moderately abundant on their home range [33]. This suggests that both would have a specialized and less diverse gut microbiome (see [34]), and might also explain why the survival of *Bradypus* (but not *Choloepus*) in captivity is so low [35]. Finally, both the three-toed sloth and koala possess low BMR and body temperatures (lower in *Bradypus*), which can result in lower rates of CH_4_ production by reducing the fermentation activity of gastrointestinal symbionts [28]. In marked contrast to the three-toed sloth, the two-toed sloth, *Choloepus* has a larger 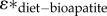 than expected for its body mass. Major physiological differences between these two species could account for the substantial observed differences. For instance, although both species have fluctuating body temperatures in phase with that of their environments, *Bradypus* shows an even more variable and lower overall daily body temperature than *Choloepus* [33,36]. In addition, although the field-measured metabolic rates of both sloths are the lowest of all non-hibernating mammals [36], both basal and field-measured metabolic rates are significantly lower in *Bradypus* (by one-third) than in *Choloepus* [36,37]. Moreover, unlike the three-toed sloth, which in the zoo was fed with a monospecific diet (sprouts of the rubber plant, *Ficus elastica*), the food fed to the two-toed sloth included spinach and other greens, quinoa, sweet potatoes, carrots and pellets of Purina Dog Chow® (table 1; electronic supplementary material). In the wild, the diet of *Choloepus* also is more diverse than that of *Bradypus*, even including components of animal origin [38–40]. Consistent with a more diverse diet, *Choloepus* also has a more variable and diverse gut microbiota than *Bradypus* [34]. Both a more diverse microbiome and a diet richer in fibre and fermentable carbohydrates seem to increase fermentation rates [28,34,41] and would explain the much higher 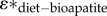 values in *Choloepus* than in *Bradypus*, both absolutely and for that expected relative to body size for the correlation observed across mammals. Finally, an experimental study of *Choloepus* showed an unexpectedly high level of methane production in this species (higher than in non-ruminant herbivores of similar body mass), explained as owing to long particle mean retention times [13]. If so, this suggests that the mean retention times in the other extant sloth, *Bradypus,* is shorter.

Most artiodactyls and perissodactyls herein had 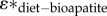 closely correlated with their body masses [42]. The only extant ungulate with a considerably higher than expected 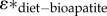 value relative to its body mass was the cow, *Bos taurus*. In general, roughage-eating ruminants have the most complex digestive tract of all mammalian herbivores, with a well-developed forestomach, small caecum and large populations of microorganisms in the rumen [43,44]. This type of ruminant digestive system involves an increase in time and capacity of fermentation, resulting in greater amounts of methane produced than a similarly sized non-ruminant, and a consequent expectation of higher 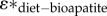 value. In fact, the dietary energy lost through extensive microbial methane production in ruminants is the highest reported for modern mammals [43]. Although precise controlled feeding data are lacking for a number of ungulates in this study, at around 100 kg regression models for foregut versus hindgut fermenters intersect with 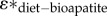 values of approximately 13‰. For mammals above approximately 200 kg, our model predicts higher 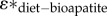 values for foregut (including ruminants) compared to hindgut fermenters.

The African elephant, *Loxodonta afric*ana, shows a slightly lower 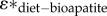 than expected for its body mass. As mixed-feeder hindgut fermenters, elephants have faster digestive throughputs than ruminants, which results in less CH_4_ production (l day^−1^) per unit body mass [31]. Strikingly, although the giant ground sloth *M. darwinii* has an estimated body mass (approx. 1600 kg) only half that of an elephant, it possesses an even higher 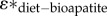 value, implying substantially higher methanogenic production. Because modern sloths carry out foregut fermentation and phylogenetically bracket all other sloths, it is most parsimonious to infer that fossil sloths such as *Mylodon* also had a foregut-fermenting digestive tract. This type of digestive strategy would explain the high 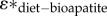 of *Mylodon*, as now expected given the steeper slope of the regression model for foregut fermenters compared to hindgut fermenters. In fact, our regression model for foregut fermenters alone predicts a higher 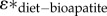 than expected for hindgut fermenters, even excluding *Mylodon* from the analysis (figure 2). Ground sloths, the largest mammals that ever existed in South America would be, together with modern hippos, the largest known non-ruminant foregut fermenters. Experimentally controlled 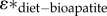 data for modern hippos are not available, but our analyses across a wide range of mammals predict that hippos will show high 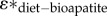 values, at least comparable to those obtained for *Mylodon*. Future studies will test if our predictions hold true and to what degree, if any, the combination of low rates of metabolism combined with amphibious lifestyles, diets of different nutritional quality and fibre content influences the expected 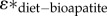 in non-ruminant foregut fermenters such as hippos and other fossil giant sloths beyond *Mylodon*.

The predicted higher 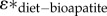 in foregut fermenters compared to hindgut fermenters across all mammalian herbivores analysed is consistent with the considerable differences of CH_4_ production observed in foregut versus hindgut fermenters [42]. However, methanogenesis does not explain the body mass effect observed in 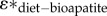 because there is an isometric relationship between body mass and methane production [42]. This new body mass effect discovery thus requires a different, as yet unknown, size-dependent mechanism to explain diet–bioapatite C-enrichment. Therefore, although methanogenesis plays a role in differences of 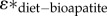 between digestive system fermentation types, it cannot explain the size effect of the relationship within each group or across all mammalian herbivores.

## Significance of implementing body mass as predictor of 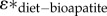


5.

Because the standard value of 14‰ of diet–bioapatite enrichment has been widely applied across all mammals since the pioneering study of Cerling & Harris [3], with some notable exceptions (see above), our new equations incorporating the body mass dependency for 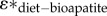 most probably will change many previous reconstructions of dietary δ^13^C of mammals, particularly at the extremes of body masses: small-bodied and megaherbivore mammals. Initial examples of the use of these new constraints can be drawn from the literature, in which previous ecological interpretations using a 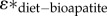 of 14‰ lead to unreasonable dietary δ^13^C values. For instance, the dwarf antelope, *Neotragus batesi*, is a small (2–3 kg) artiodactyl [45] restricted to dense forests of tropical Africa. Dental δ^13^C data of *N. batesi* from the Ituri Forest showed an average signature of −25.6‰. The use of the 14‰ 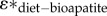 enrichment reconstructed the δ^13^C of the plant diet for this species as −39.6‰, lower than any plant ever recorded in that forest (δ^13^C of subcanopy plants at Ituri range between −31‰ and −36.5‰ [9]). Incorporating our new body mass-dependent enrichment, *N. batesi*, a small foregut fermenter, would show an average 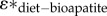 value of 10.86‰, which indicates a plant diet with δ^13^C values of −36‰, a δ^13^C signature observed for several plants sampled in that forest [9].

Recognition of the body mass dependence of carbon isotope enrichment in mammals is essential for accurate understanding of the relative δ^13^C differences among members of a herbivore community, which are used to reconstruct feeding niches and environments, and to evaluate ecological and environmental changes through time. Thus, species with similar δ^13^C values, which would be interpreted as overlapping in resource usage when using a single standard 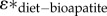 value, could reveal distinct niche partitioning if body mass is taken into account. Similarly, imprecise or even misleading reconstructions of dietary changes through time can arise if the δ^13^C of the plants consumed have been inferred with a single 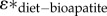 value for all herbivores. For instance, application of our proposed body mass-corrected 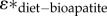 could refine determination of the precise time when hominins began incorporating C_4_ plants in their diets and the proportion that these represented in the evolution of hominin diets over time. Controlled feeding experiments may reveal the same body mass-specific enrichment between diet and other tissues, and this differential isotopic enrichment also could prove valid in non-mammalian organisms once tested through the same experimental methods.

Another important impact of our study would be on mean annual precipitation (MAP) calculations founded on δ^13^C values of the plant community of the area under study [46]. Because the estimated δ^13^C of the palaeofloral community is indirectly inferred and calculated from δ^13^C of dental bioapatite in fossil mammalian herbivores [46,47], a body mass correction of the 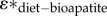 can result in different δ^13^C vegetational values and a subsequent different MAP estimation. Accounting for the body mass influence on enrichment can profoundly alter some reconstructions of plant communities and their implied MAP values.

As the value of 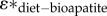 is related to digestive physiology, it can reveal physiological aspects in species that were not possible to assess previously, such as the extent of microbial fermentation to break up the ingested food, diet quality and type of gastric fermentation (foregut versus hindgut). For example, our analyses indicate that the giant ground sloth *Mylodon* was a highly methanogenic foregut fermenter. *Mylodon*, a narrow-muzzled sloth interpreted as a selective feeder based on snout anatomy [48], is now documented as the mammalian herbivore with the largest known 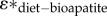 value; therefore, we would expect other Quaternary fossil sloths that are twice as large as *Mylodon* (e.g. *Megatherium americanum, Eremotherium* spp*.*) to show even larger 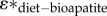 values, especially if they were bulk feeders, as suggested for the giant ground sloth, *Lestodon armatus* [48]. Sloths challenge previously proposed ideas of constraints imposed by large body sizes on foregut fermentation [49], showing an unparalleled combination of morphological variability and physiological traits probably responsible for their extremely wide ecological diversification in the Americas.

## Supplementary Material

ESM for 'Body mass predicts isotope enrichment in herbivorous mammals' by Julia Tejada et al.

## Data accessibility

All data supporting this manuscript are available in the electronic supplementary material.
