# Supplementary material for: Body mass predicts isotope enrichment in herbivorous mammals
Source: Proc Biol Sci. 2018 Jun 27;285(1881):20181020. doi: 10.1098/rspb.2018.1020 (PMC6030519; doi:10.1098/rspb.2018.1020)
Supplement: ESM for 'Body mass predicts isotope enrichment in herbivorous mammals' by Julia Tejada et al. [file rspb20181020supp1.pdf]

ELECTRONIC SUPPLEMENTARY MATERIALS FOR:

"BODY MASS PREDICTS ISOTOPE ENRICHMENT IN HERBIVOROUS MAMMALS"

Authors: Julia V. Tejada-Lara[1,2,3]*, Bruce J. MacFadden[4], Lizette Bermudez[5], Gianmarco Rojas[5], Rodolfo Salas-Gismondi[2,3,6], John J. Flynn[2]

Affiliations:

[1]Department of Earth and Environmental Sciences, Lamont-Doherty Earth Observatory, Columbia University. New York.

[2]Division of Paleontology, American Museum of Natural History. New York.

[3]Departamento de Paleontología de Vertebrados, Museo de Historia Natural, Universidad Nacional Mayor de San Marcos. Lima, Perú.

[4]Florida Museum of Natural History, University of Florida. Gainesville, Florida.

[5]Zoológico de Huachipa. Lima, Perú.

[6]BioGeoCiencias Lab, Facultad de Ciencias y Filosofía/CIDIS, Universidad Peruana Cayetano Heredia, Lima, Perú

*Corresponding author

*Correspondence to: jtejada@ldeo.columbia.edu or jtejada@amnh.org

MATERIALS AND METHODS

Stable isotope analyses

Samples of teeth and feces of *Bradypus variegatus* and *Choloepus hoffmanni* were collected from adult individuals raised at the Huachipa Zoo (Lima, Peru), except for one dental sample of *Choloepus hoffmanni* from the Tampa's Lowry Park Zoo (Florida, USA). Individuals were either born at or adopted by the zoos at a young age, and spent several years being fed a controlled diet (spanning 5 to 12 years under a controlled diet feeding regime at the zoo). Samples of living specimens from the Huachipa Zoo were collected by the zoo staff during the annual health inspection. Dental bioapatite was analyzed for $\delta^{13}C$. *Choloepus* and *Bradypus* have ever-growing teeth which in captivity are not sufficiently worn down over time as they are in natural conditions. During health inspections at zoos, excessively grown caniniforms (*Choloepus*) and incisiviforms (*Bradypus*) are trimmed to prevent animals from hurting themselves (in a similar fashion to veterinarian treatments for hamsters). Instead of being discarded, these disposable samples of dental tissues were collected and used for isotopic analyses. Samples were later powdered in the lab using a mortar and pestle.

Powdered samples of dental bioapatite were analyzed for $\delta^{13}C$ and $\delta^{18}O$. Modern samples were first treated for three hours with H_2O_2 to remove organic contaminants, rinsed three times with distilled water, bathed for an hour in 0.1 N acetic acid to remove any secondary carbonates, rinsed three more times with distilled water followed by a fourth rinse with methanol, and finally dried. Samples were run on a Finnigan MAT 252 isotope ratio mass spectometer (IRMS) with a Kiel device. Foodstuff and feces were collected on a daily basis for 11 and 15 days respectively, and analyzed for $\delta^{13}C$ and $\delta^{15}N$. All organic samples were freeze dried and homogenized in a Spex 67000 liquid nitrogen mill. Weight %C and %N were determined using a Carlo-Erba 1500 elemental analyzer after IRMS analysis. All the samples were processed and analyzed at the Department of Geological Sciences, University of Florida (Gainesville).

Fossil dung was freeze dried, homogenized, and processed as for the modern organic samples. Teeth of fossil sloths were sampled using a Dremel® drill and carbide dental burrs. Approximately 0.1 g of powdered outermost dentine was collected and processed as described above.

Values are reported using the conventional permil (‰) notation wherein $\delta^{13}C = (R_{sample}/R_{standard} - 1) \times 1000)$, and $R={}^{13}C/{}^{12}C$. Values of $\delta^{13}C$ are reported relative to the VPDB standard. Isotope enrichment (ε^*) was calculated as in Cerling and Harris [3], i.e., as a function of α^* (apparent fractionation), as follows: $\varepsilon^* = (\alpha^* - 1) \times 1000$, where $\alpha^* = (1000 + \delta^{13}C_{tissue})/(1000 + \delta^{13}C_{diet})$. The superscript (*) indicates that isotopic equilibrium is not assumed.

Analytical precision of standards:
Inorganic samples (teeth bioapatite, NBS-19 standard)
$\delta^{13}C = 0.02$; $\delta^{18}O = 0.05$
Organic samples (food and feces; USGS-40 standard):
$\delta^{13}C = 0.09$; $\delta^{15}N = 0.22$

Foodstuff

A concentration-weighted linear mixing model [49] was used to calculate the isotopic signatures of any mixed diet. In this model, the proportional contribution of each component to the mixture (and therefore to the consumer) equals the fraction of that component in the mixture multiplied by its elemental concentration (in this case, wt %C and wt %N) and divided by the sum of the products of fraction and concentration of each dietary component, as follows:

$$f_{X,C} = \frac{f_{X,B}[C]_X}{f_{X,B}[C]_X + f_{Y,B}[C]_Y + f_{Z,B}[C]_Z}$$

$$f_{Y,C} = \frac{f_{Y,B}[C]_Y}{f_{X,B}[C]_X + f_{Y,B}[C]_Y + f_{Z,B}[C]_Z}$$

$$f_{Z,C} = \frac{f_{Z,B}[C]_Z}{f_{X,B}[C]_X + f_{Y,B}[C]_Y + f_{Z,B}[C]_Z}$$

Where $f\,(\text{x, C})$; $f\,(\text{y, C})$, $f\,(\text{z, C})$, are the fractions of assimilated C, of components “x”, “y”, “z” in the mixed diet.

Regression models and traitgram

Data for linear regression models of mammals other than sloths, were collected from the literature (table 2, table S1). Values of $\varepsilon^*_{\text{diet-bioapatite}}$ were regressed against body mass (BM), basal rate of metabolism (BMR), average rectal temperature, and range of rectal temperature variation. All data were log transformed (ln). Statistics and regression modeling were performed with RStudio (v 1.0.136) using packages MASS, foreign, quantreg, MuMIn, and lme4. Traitgram was performed with the R package phytools [50]. We assessed relationships between variables by performing OLS, quantile, and robust regression models; Cook’s distance was used as an estimate for the influence of outliers. Linear mixed effect model analyses based on AICc tests were used to assess whether a single variable, reduced model, or various combinatorial model subsets were better supported by the data than a complete global model that includes all the potential predictors. Tree topology for Figure 1 is from O’Leary et al. [51]. Divergence ages (in Ma, or Megannum), only for illustrative purposes (providing approximations for visual representation of the pattern of lineage splitting, but not used in calculations), are mean estimates from the literature (see below).

Regression formulae

This paper proposes three regression formulae (body-mass calibrated) to calculate the C diet-bioapatite enrichment ($\varepsilon^*_{\text{diet-bioapatite}}$) of herbivorous mammals (both extant and extinct). The first formula (A) is based on the regression analysis of correlation of all mammals included:

$$\boldsymbol{\varepsilon * = 2.4 + 0.034\ (\mathrm{BM})} \qquad \textbf{(A)}$$

Where BM is the body mass in kg and should be log transformed (ln). The obtained diet-bioapatite ε* will need to be inverted (e^x) to obtain the ‰ value to be applied for interpretation of the isotopic signature of the herbivorous mammal under study.

This formula (A) should be used when the type of digestive fermentation of the mammal under study is unknown, does not fall within foregut or hindgut types of fermentation (e.g. giant panda), or to get a general or most conservative body-size calibrated value for $\varepsilon^*_{\text{diet-bioapatite}}$.

The two other formulae we propose separate mammals by foregut (B) versus hindgut fermenters (C):

$$\boldsymbol{\varepsilon * = 2.34 + 0.05\ (BM)} \qquad \textbf{(B)}$$

$$\boldsymbol{\varepsilon * = 2.42 + 0.032\ (BM)} \qquad \textbf{(C)}$$

These formulae should be applied when the type of digestive fermentation (foregut vs hindgut) is known or most likely given phylogenetic history of the mammal under study. As for the first formula, BM is in kg and log transformed (ln), and ε* needs to be inverted (e^x) to obtain the ‰ value to be applied for interpretation of the isotopic signature of the herbivore mammal under study. Table 3 documents that multiple models agree closely in terms of regression slopes and intercepts. Rather than select one arbitrarily, we advocate instead for applying a model averaging approach. As discussed by Sears et al. [52], model averaging is appropriate when there is no single, clearly identifiable "correct" model, and given the close agreement across all of the models and that each performs equally well in estimating the variables, use of the mean of the individual estimates is appropriate here.

Sources of divergence ages

Bradypus / Choloepus+Mylodon: 27 ± 3 Ma [53]; or 29.3 Ma sensu [54].
Mylodon / Choloepus: 27.5 Ma [54].
Muridae (*Mus*) / Cricetidae (*Microtus*): 65.8 Ma [55].
Rabbit / Mouse: 65.5 Ma; based on *Mimotona wana* from [51]
Giraffidae / remaining Pecora (i.e. including *Bos*): 19 Ma [56].
Suidae / Ruminantia: 35.2 Ma; based on *Elomeryx crispus* from [51].
Ruminantia / Camelidae: 49-55 Ma; (based on *Pseudamphimeryx*, first ruminant, [57] from [58]).
Camelus / Lama: 25 Ma [59].
Equus / Diceros: 52-58 Ma; split Ceratomorpha and Hippomorpha [60].
Equus caballus / Equus burchelli: 3 Ma [61].
Arytiodactyla / Perissodactyla: 55.4-50.3 Ma, based on *Hyracotherium angustidens* [51].
Carnivora / Eungulata: 84.9 Ma [62].
Afrotheria: 101 Ma [62].
Euarchontoglires / Laurasiatheria: 98.9 Ma [62].
Xenarthra / Epitheria: 101 Ma [62].
Marsupialia / Eutheria: 147.7 Ma [62].

Sources of life history trait values

BM: body mass; BMR: basal metabolic rate, and basal metabolic rate corrected for body mass using the Kleiber value; Temp: body temperature, as average rectal temperature and breadth of range of rectal temperature. † = extinct taxon. Unpublished data from [6] provided by the lead author of that study, B. Passey.

Choloepus hoffmanni
BM: data from this study; BMR [35]; Temp: data from this study and [36].
Notes: Range of temperature is from [36] but average temperature is from specimens analyzed in this study (Huachipa Zoo).

Bradypus variegatus
BM: data from this study; BMR [35], Temp: data from this study and [36].
Notes: same as for *Choloepus*.

†Mylodon darwinii
BM [63].
Note: Although McNab (1985, [35]) provided estimates for BMR and BMR/Kleiber value for *Mylodon listai*, we consider these estimations as unreliable because they are based on highly questionable foundations (body hair length as a proxy for thermal insulation correlated to BMR).

Bos taurus
BM [unpublished data from 6]; BMR [64]; Temp [65].

Sus scrofa
BM [unpublished data from 6]; BMR [64]; Temp [66].

Oryctolagus cuniculus
BM [unpublished data from 6]; BMR [67]; Temp [68].
Notes: BMR based on a close relative, *Lepus alleni*.

Microtus ochrogaster
BM [unpublished data from 6]; BMR [69]; Temp [69,70].
Notes: BMR based on a congener, *Microtus guentheri*.

Mus musculus
BM [5]; BMR [64]; Temp [71,72]

Lama guanicoe
BM [73]; BMR [73,74]; Temp [75].
Notes: BMR based on a congener, *Lama glama*.

Giraffa camelopardalis
BM [76]; BMR [77]; Temp [78].
Notes: BMR calculated from resting metabolic rate (RMR). BMR was assumed to account for ~85% of the RMR.

Diceros bicornis
BM [76,79]; Temp [80].

Camelus bactrianus
BM [67,76,79]; BMR [67]; Temp [81].
Notes: BMR based on a congener, *Camelus dromedarius*.

Equus caballus
BM [82]; BMR [64]; Temp [83].

Equus burchelli
BM [84]; BMR [64]; Temp [84].
Notes: BMR based on a congener, *E. caballus*.

Ailuropoda melanoleuca
BM [85]; BMR [85]; Temp [86].
Notes: BMR calculated from resting metabolic rate.

Loxodonta africana
BM [82]; BMR [64]; Temp [87].
Notes: BMR based on a close relative, *Elephas maximus*

Phascolarctos cinereus
BM [67,82]; BMR [67]; Temp [88].

TAXA	E*(mean)	BM (Kg)	FERM	BMR	BMR/Kleiber value	Temp Mean	Temp Variation	Temp Range
Choloepus hoffmanni	12.6	8.28	fore	0.188	0.44	34.4	33.4-36.2	2.8
Bradypus variegatus	10.3	4.23	fore	0.181	0.42	31	28.4-37.6	9.2
Mylodon darwinii†	15.6	1600	fore	NA	NA	NA	NA	NA
Bos taurus	14.6	322.7	fore	0.17	1.14	38.7	38.3-39.1	0.8
Sus scrofa	13.3	128.6	hind	0.11	0.53	39.3	38.8-39.8	1
Oryctolagus cuniculus	12.8	3.5	hind	0.45	1	39.3	38.1-40.8	2.7
Microtus ochrogaster	11.5	0.05	hind	1.18	0.92	38	37.3-38.7	1.4
Mus musculus	9.1	0.021	hind	3.4	2.12	37.65	36-39.3	3.3
Lama guanicoe	12.9	120	fore	0.23	1.24	38	37.5-38.5	1
Giraffa camelopardalis	14.1	1500	fore	0.13	1.08	38.5	38-39	1
Diceros bicornis	14.4	1089	hind	NA	NA	37.74	36.8-38.6	1.8
Camelus bactrianus	13.7	454	fore	0.1	0.74	37	34-40	6
Equus caballus	13.7	260	hind	0.25	1.65	37.7	37.2-38.2	1
Ailuropoda melanoleuca	10.1	92	NA	0.082	0.42	37.3	37-37.6	0.6
Phascolartos cinereus	10.3	4.8	hind	0.22	0.54	36	34.2-37.7	3.5
Loxodonta africana	14.3	3600	hind	0.15	1.78	36.5	36-37	1
Equus burchelli	13.2	280	hind	NA	NA	39.3	38.4-41.8	3.4

Table S1. Summary of the $\varepsilon^*_{\text{diet-bioapatite}}$ values (highlighted box) for taxa in this study and other life history trait values. Taxonomic selection was driven by: (1) accuracy of dietary $\delta^{13}C$ data and (2) sampling of phylogenetic diversity. Abbreviations: BM: body mass (kg), FERM: type of digestive system (foregut vs hindgut fermentation), BMR: basal rate of metabolism ($cm^3O_2/g*h$), Temp: body temperature (°C). Sources for all values above and in Table S2. † is a recently extinct Pleistocene sloth.

TAXA	ε*	Controlled	Source of ε*
Choloepus hoffmanni	12.62 ± 0.68	Yes	this study
Bradypus variegatus	10.31 ± 1.03	Yes	this study
Mylodon darwinii†	15.63± 0.51	Yes, (diet from dung)	this study
Bos taurus	14.6± 0.3	Yes	[6]
Sus scrofa	12.9± 0.5	Yes	[6]
Oryctolagus cuniculus	12.8± 0.7	Yes	[6]
Microtus ochrogaster	11.5± 0.3	Yes	[6]
Mus musculus	9.1± 1.6	Yes	[5]
Lama guanicoe	12.9	No	[3]
Giraffa camelopardalis	14.1	No	[3]
Diceros bicornis	14.4	No	[3]
Camelus bactrianus	13.7	Partial	[3]
Equus caballus	13.7	No	[3]
Loxodonta africana	14.3	No	[3]
Ailuropoda melanoleuca	10.1	Partial	[89]
Equus burchelli	13.2	No	[90]
Phascolartos cinereus	10.3	Partial	[91]

Table S2. Summary of the $\varepsilon^*_{diet\text{-}bioapatite}$ values included in this study. Selection of taxa was determined by: (1) dietary $\delta^{13}C$ controlled, (2) partially controlled (i.e., monospecific diets), or (3) not controlled but still with a narrow range of reported $\delta^{13}C_{diet}$ variation in the wild. † is an extinct taxon.

	AICc
Brownian Motion	-11.17615193
Lambda	-9.064478475
Delta	-10.73648238
Kappa	-8.28545515
Early Burst	-8.186953844
Ornstein Uhlenbeck	-10.79611806
White Noise	-11.93408278

Table S3. Summary of AIC analyses comparing different models of trait evolution. Lowest AICc score (gray highlight) corresponds to a White Noise model, which disregards phylogeny. The second lowest AICc score corresponds to Brownian motion (BM), which further emphasizes the lack of phylogenetic signal in the known values of $\varepsilon^*_{diet\text{-}bioapatite}$ across mammals. Data were fitted to the framework phylogenetic tree with the R package GEIGER [92]. Refer to [92] for explanation of each model of trait evolution.

ε* vs:	Body Mass		BMR		BMR/Kleiber value		Temperature (average)		Breadth of temp variation	
	R^2	*p*-value	R^2	*p*-value	R^2	*p*-value	R^2	*p*-value	R^2	*p*-value
All taxa	0.62	0.000	0.24	0.078	0.06	0.366	0.16	0.122	0.12	0.19
Foregut	0.78	0.008	0.15	0.454	0.56	0.089	0.89	0.005	0.51	0.11
Hindgut	0.74	0.003	0.56	0.054	0.00	0.975	0.07	0.482	0.36	0.085

Table S4. Summary statistics for all regression analyses. Highlighted boxes indicate significant values. Only body mass correlated significantly with ε* in all cases (i.e., in analyses with all taxa included as well as for mammals separated by type of digestive system [foregut and hindgut only]). Similar results (no significant correlations of ε* with BMR corrected for body mass, for all mammals or either digestive system subset) when applying the alternative body mass scaling function of [93]; Kleiber value remains the most widely accepted scaling function for BMR/BM covariance, and we present it here because [93] excludes all ruminants, which are a substantial number of the taxa in our analyses and prior studies of carbon isotope enrichment in dental bioapatite.

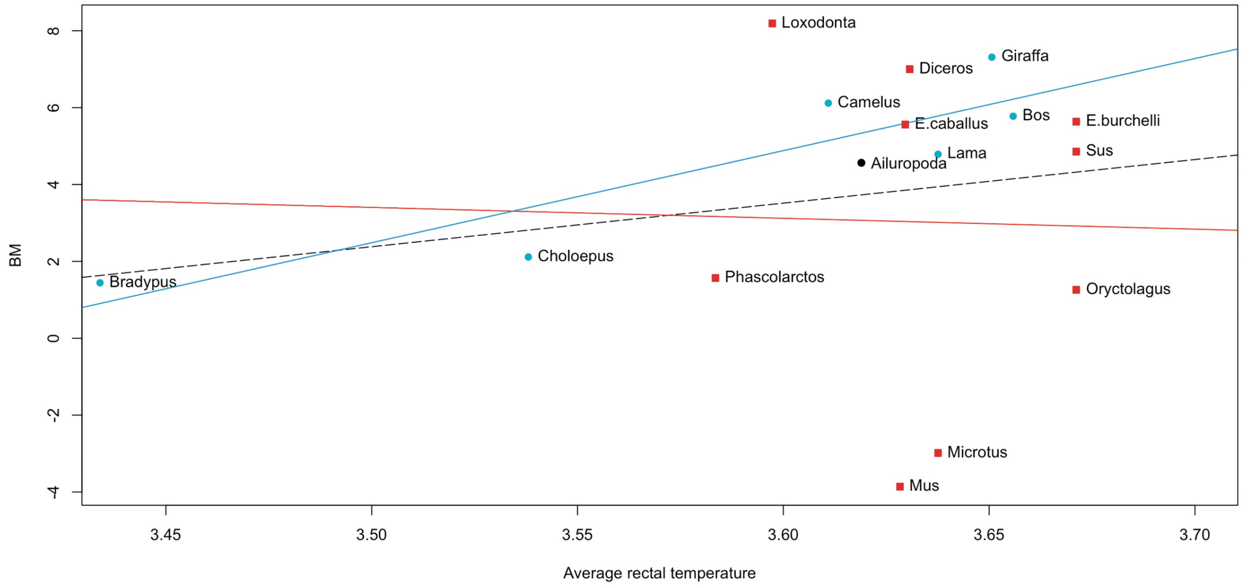

Figure S1. Correlation between body mass (BM) and average rectal temperature. Values are log transformed. All mammals (black dashed line): R^2=0.04, p-value=0.47; hindgut fermenters (red line and symbols): R^2=0.00, p-value=0.95, foregut fermenters (greenish line and symbols): R^2= 0.80, **p-value= 0.02**.

Supplementary References:

50. Revell LJ. 2011 phytools: an R package for phylogenetic comparative biology (and other things). *Methods Ecol Evol* **3**, 217–223. (doi:10.1111/j.2041-210x.2011.00169.x)
51. O'Leary MA et al. 2013 The Placental mammal ancestor and the post-K-Pg radiation of placentals. *Science* **339**, 662–667. (doi:10.1126/science.1229237)
52. Sears KE, Finarelli JA, Flynn JJ, Wyss AR. 2008 Estimating body mass in New World "monkeys" (Platyrrhini, Primates), with a consideration of the Miocene platyrrhine, *Chilecebus carrascoensis*. *American Museum Novitates* **3617**:1–29. (doi:10.1206/627.1)
53. Delsuc F, Superina M, Tilak M-K, Douzery EJP, Hassanin A. 2012 Molecular phylogenetics unveils the ancient evolutionary origins of the enigmatic fairy armadillos. *Molecular Phylogenetics and Evolution* **62**, 673–680. (doi:10.1016/j.ympev.2011.11.008)
54. Slater GJ, Cui P, Forasiepi AM, Lenz D, Tsangaras K, Voirin B, de Moraes-Barros N, MacPhee RDE, Greenwood AD. 2016 Evolutionary relationships among extinct and extant sloths: the evidence of mitogenomes and retroviruses. *Genome Biology and Evolution* **8**, 607–621. (doi:10.1093/gbe/evw023)
55. Hedges SB, Kumar S. 1998 A molecular timescale for vertebrate evolution. *Nature* **392**, 917–920. (doi:10.1038/31927)
56. Bibi F. 2013 A multi-calibrated mitochondrial phylogeny of extant Bovidae (Artiodactyla, Ruminantia) and the importance of the fossil record to systematics. *BMC Evolutionary Biology* **13**, 166. (doi:10.1186/1471-2148-13-166)
57. Savage DE, Russell DE. 1983 Mammalian paleofaunas of the world. Addison-Wesley, London.
58. Theodor JM. 2004 Clock divergence estimates and the fossil record of Cetartiodactyla. *J. Paleo.* **78**, 39–44. (10.1666/0022-3360(2004)078<0039:MCDEAT>2.0.CO;2)
59. Cui P, Ji R, Ding F, Qi D, Gao H, Meng H, Yu J, Hu S, Zhang H. 2007 A complete mitochondrial genome sequence of the wild two-humped camel (*Camelus bactrianus ferus*): an evolutionary history of camelidae. *BMC Genomics* **8**, 241. (doi:10.1186/1471-2164-8-241)
60. Steiner CC, Ryder OA. 2011 Molecular phylogeny and evolution of the Perissodactyla. *Zoological Journal of the Linnean Society* **163**, 1289–1303. (doi:10.1111/j.1096-3642.2011.00752.x)
61. Ishida N, Oyunsuren T, Mashima S, Mukoyama H, Saitou N. 1995 Mitochondrial DNA sequences of various species of the genus *Equus* with special reference to the phylogenetic relationship between Przewalskii's wild horse and domestic horse. *Journal of Molecular Evolution* **41**, 180–188. (doi:10.1007/bf00170671)
62. Bininda-Emonds ORP et al. 2007 The delayed rise of present-day mammals. *Nature* **446**, 507–512. (doi:10.1038/nature05634)
63. Vizcaíno SF, Bargo MS, Cassini GH. 2006 Dental occlusal surface area in relation to body mass, food habits and other biological features in fossil xenarthrans. *Ameghiniana* **43**,11–26.

64. Eisenberg JF. 1981 *The mammalian radiation: An analysis of trends in evolution, adaptation, and behavior*. U. Chicago Press, Chicago.

65. Espinoza JL, Sanchez J, Gracia JA, Sanchez JR, Ortega R, Palacios A. 2009 Thermoregulation differs in Chinampo (*Bos taurus*) and locally born dairy cattle. Turkish *Journal of Veterinary and Animal Sciences* **33**, 175–180.

66. Jara AL, Hanson JM, Gabbard JD, Johnson SK, Register ET, He B, Tompkins SM. 2016 Comparison of Microchip Transponder and Noncontact Infrared Thermometry with Rectal Thermometry in domestic swine (*Sus scrofa domestica*). *Journal of the American Association for Laboratory Animal Science* **55**, 588–593.

67. McNab BK. 1986 The Influence of Food Habits on the Energetics of Eutherian Mammals. *Ecological Monographs* **56**, 1–19. (doi:10.2307/2937268)

68. Chen PH, White CE. 2006 Comparison of rectal, microchip transponder, and infrared thermometry techniques for obtaining body temperature in the laboratory rabbit (*Oryctolagus cuniculus*). *Journal of the American Association for Laboratory Animal Science* **45**, 57–63.

69. Banin D, Haim A, Arad Z. 1994 Metabolism and thermoregulation in the Levant vole *Microtus guentheri*: The role of photoperiodicity. *Journal of Thermal Biology* **19**, 55–62. (doi:10.1016/0306-4565(94)90009-4)

70. Cherry RH, Verner L. 1975 Seasonal Acclimatization to Temperature in the Prairie Vole, *Microtus ochrogaster*. *American Midland Naturalist* **94**, 354. (doi:10.2307/2424431)

71. Hudson JW, Scott IM. 1979 Daily Torpor in the Laboratory Mouse, *Mus musculus* var. Albino. *Physiological Zoology* **52**, 205–218. (doi:10.1086/physzool.52.2.30152564)

72. Rhodes JS, Koteja P, Swallow JG, Carter PA, Garland T Jr. 2000 Body temperatures of house mice artificially selected for high voluntary wheel-running behavior: repeatability and effect of genetic selection. *Journal of Thermal Biology* **25**, 391–400. (doi:10.1016/s0306-4565(99)00112-6)

73. Hayssen V, Lacy RC. 1985 Basal metabolic rates in mammals: Taxonomic differences in the allometry of BMR and body mass. *Comparative Biochemistry and Physiology Part A: Physiology* **81**, 741–754. (doi:10.1016/0300-9629(85)90904-1)

74. El-Nouty F., Yousef M., Magdub A., Johnson H. 1978 Thyroid hormones and metabolic rate in burros, *Equus asinus*, and llamas, *Lama glama*: Effects of environmental temperature. *Comparative Biochemistry and Physiology Part A: Physiology* **60**, 235–237. (doi:10.1016/0300-9629(78)90238-4)

75. de Lamo DA, Lacolla D, Heath JE. 2001 Sweating in the guanaco (*Lama guanicoe*). *Journal of Thermal Biology* **26**, 77–83. (doi:10.1016/s0306-4565(00)00014-0)

76. Nowak RM. 1999 Walker's Mammals of the World, vol. 1. JHU Press.

77. Langman VA, Bamford OS, Maloiy GMO. 1982 Respiration and metabolism in the giraffe. *Respiration Physiology* **50**, 141–152. (doi:10.1016/0034-5687(82)90013-5)

78. Mitchell G, Skinner J. 2004 Giraffe thermoregulation: a review. *Transactions of the Royal Society of South Africa* **59**, 109–118. (doi:10.1080/00359190409519170)

79. AnAge database www.genomics.senescence.info

80. Morkel P vdB et al. 2012 Serial temperature monitoring and comparison of rectal and muscle temperatures in immobilized free-ranging black rhinoceros (*Diceros bicornis*). *Journal of Zoo and Wildlife Medicine* **43**, 120–124. (doi:10.1638/2011-0009.1)

81. Lensch J. 1999 The two-humped camel (*Camelus bactrianus*). FAO Corporate Document Repository. ISSN 1014-6954.

82. Elgar MA, Harvey PH. 1987 Basal metabolic rates in mammals: allometry, phylogeny and ecology. *Functional Ecology* **1**, 25. (doi:10.2307/2389354)

83. Holcomb KE. 2012 Behavioral and physiological responses of domestic horses to provision of shade in a hot, sunny environment. PhD Thesis. University of California, Davis.

84. Grubb P. 1981 *Equus burchelli*. Mammalian Species, 1. (doi:10.2307/3503962)

85. Fei Y, Hou R, Spotila JR, Paladino FV, Qi D, Zhang Z. 2016 Metabolic rates of giant pandas inform conservation strategies. *Scientific Reports* **6**. (doi:10.1038/srep27248)

86. Jin Y, Qiao Y, Liu X, Pu T, Xu H, Lin D. 2016 Immobilization of wild giant panda (*Ailuropoda melanoleuca*) with dexmedetomidine–tiletamine–zolazepam. *Veterinary Anaesthesia and Analgesia* **43**, 333–337. (doi:10.1111/vaa.12301)

87. Kinahan AA, Inge-moller R, Bateman PW, Kotze A, Scantlebury M. 2007 Body temperature daily rhythm adaptations in African savanna elephants (*Loxodonta africana*). *Physiology & Behavior* **92**, 560–565. (doi:10.1016/j.physbeh.2007.05.001)

88. Adam D et al. 2016 Surgical implantation of temperature-sensitive transmitters and data-loggers to record body temperature in koalas (*Phascolarctos cinereus*). *Australian Veterinary Journal* **94**, 42–47. (doi:10.1111/avj.12393)

89. Han H, Wei W, Nie Y, Zhou W, Hu Y, Wu Q, Wei F. 2016 Distinctive diet-tissue isotopic discrimination factors derived from the exclusive bamboo-eating giant panda. *Integr. Zoo.* **11**, 447–456. (10.1111/1749-4877.12208)

90. Bocherens H, Koch PL, Mariotti A, Geraads D, Jaeger J-J. 1996 Isotopic Biogeochemistry (^{13}C, ^{18}O) of Mammalian Enamel from African Pleistocene Hominid Sites. *Palaios* **11**, 306–318. (doi:10.2307/3515241)

91. Fraser RA. 2005 A study of stable carbon, nitrogen and oxygen isotopes in modern Australian marsupial herbivores, and their relationships with environmental conditions. Australian National University, Canberra.

92. Harmon LJ, Weir JT, Brock CD, Glor RE, Challenger W. 2007 GEIGER: investigating evolutionary radiations. *Bioinformatics* **24**, 129–131. (doi:10.1093/bioinformatics/btm538)

93. White CR, Seymour RG. 2003 Mammalian basal metabolic rate is proportional to body mass$^{2/3}$. *Proceedings of the National Academy of Sciences, USA* **100**, 4046-4049.
